# Supplementary material for: Deep cryptic diversity in the Craugastor podiciferus Species Group (Anura: Craugastoridae) of Isthmian Central America revealed by mitochondrial and nuclear data
Source: PeerJ. 2025 Jan 17;13:e18212. doi: 10.7717/peerj.18212 (PMC11745134; doi:10.7717/peerj.18212)
Supplement: Supplemental Information 1 — Institutional voucher number (or field collector number) and locality information for the specimens used in molecular phylogenetic analyses. Museum collection acronyms follow Frost (2023), with the addition of the Círculo Herpetológico de Panamá (CH). Field collector numbers refer to Andrew J. Crawford (AJC), Erick Arias (EAP), and the Costa Rica Amphibian Research Center private collection (CRARC). [file peerj-13-18212-s001.docx]

| ID | Species | Institutional vouchers | Collection locality | Elevation (m) | Geographic coordinates | | GenBank Accession Number | | |
| --- | --- | --- | --- | --- | --- | --- | --- | --- | --- |
|  |  |  |  |  | Latitude | Longitude | 16S | COI | ddRAD |
|  | *Craugastor loki* | IBH:30460 | Santa María Chimalapa, Oaxaca, MX | 183 | 17.154 | -94.236 | OR419510 | OR420802 | ––– |
|  | *Craugastor rhodopis* | GP 2098 | Acajete, Veracruz, MX | 44 | 19.63 | -97.012 | ––– | ––– | SAMN43761706 |
|  | *Craugastor aenigmaticus* clade | | | | | |  |  |  |
| 1 | *Craugastor aenigmaticus* | SMF 104020 | Changuinola, Bocas del Toro, PA | 2388 | 8.9139 | -82.7088 | MK211615 | MK211577 | SAMN43761707 |
| 2 | *Craugastor aenigmaticus* | UCR 21951 | Telire, Talamanca, CR | 2700 | 9.3488 | -83.1750 | MK211616 | MK211578 | SAMN43761708 |
| 3 | *Craugastor aenigmaticus* | UCR 22737 | Buenos Aires, Puntarenas, CR | 2660 | 9.3224 | -83.2028 | MK211617 | MK211579 | SAMN43761709 |
|  | *Craugastor podiciferus* clade | | | | | |  |  |  |
| 4 | *Craugastor sagui* | SMF 104014 | Nole Duima, Ngöbe Buglé, PA | 1762 | 8.5571 | -81.8245 | MK211623 | ––– | SAMN43761710 |
| 5 | *Craugastor sagui* | SMF 104015 | Nole Duima, Ngöbe Buglé, PA | 1700 | 8.4997 | -81.7724 | MK211624 | MK211580 | SAMN43761711 |
| 6 | *Craugastor zunigai* | UCR 20389 | Buenos Aires, Puntarenas, CR | 1500 | 9.1112 | -83.1006 | MK211625 | MK211581 | ––– |
| 7 | *Craugastor zunigai* | UCR 22709 | Coto Brus, Puntarenas, CR | 1980 | 8.9751 | -82.8243 | MK211626 | MK211582 | SAMN43761712 |
| 8 | *Craugastor blairi* | FMNH 257562 | Gualaca, Chiriquí, PA | 1000 | 8.7500 | -82.217 | ––– | ––– | SAMN43761713 |
| 9 | *Craugastor blairi* | SMF 104023 | Gualaca, Chiriquí, PA | 1280 | 8.6781 | -82.2101 | MK211628 | MK211584 | ––– |
| 10 | *Craugastor blairi* | SMF 102024 | Gualaca, Chiriquí, PA | 1730 | 8.6775 | -82.1980 | MK211627 | MK211583 | ––– |
| 11 | *Craugastor blairi* | SMF 104027 | Bugaba, Chiriquí, CR | 2134 | 8.8494 | -82.5154 | MK211629 | MK211585 | ––– |
| 12 | *Craugastor podiciferus* | CRARC 0012 | Turrialba, Cartago, CR | 2250 | 10.0192 | -83.7132 | MK211633 | MK211589 | ––– |
| 13 | *Craugastor podiciferus* | EAP 0536 | El Guarco, Cartago, CR | 2395 | 9.7126 | -83.9488 | ––– | ––– | SAMN43761714 |
| 14 | *Craugastor podiciferus* | EAP 0810 | Talamanca, Limón, CR | 1860 | 9.3659 | -83.0417 | MK211640 | MK211596 | ––– |
| 15 | *Craugastor podiciferus* | SMF 104005 | Changuinola, Bocas del Toro, PA | 1766 | 8.9908 | -82.6716 | MK211641 | MK211597 | ––– |
| 16 | *Craugastor podiciferus* | UCR 19853 | Telire, Talamanca, CR | 1817 | 9.3580 | -83.2294 | MK211639 | MK211595 | ––– |
| 17 | *Craugastor podiciferus* | UCR 19856 | Telire, Talamanca, CR | 1817 | 9.3580 | -83.2294 | MK211637 | MK211593 | ––– |
| 18 | *Craugastor podiciferus* | UCR 19860 | Telire, Talamanca, CR | 2108 | 9.3645 | -83.2164 | MK211636 | MK211592 | SAMN43761715 |
| 19 | *Craugastor podiciferus* | UCR 19862 | Telire, Talamanca, CR | 2108 | 9.3645 | -83.2164 | MK211638 | MK211594 | ––– |
| 20 | *Craugastor podiciferus* | UCR 20992 | Alfaro Ruiz, Alajuela, CR | 2143 | 10.2272 | -84.3482 | MK211632 | MK211588 | ––– |
| 21 | *Craugastor podiciferus* | UCR 22146 | Vázquez de Coronado, San José, CR | 1700 | 10.0263 | -83.9448 | MK211635 | MK211591 | ––– |
| 22 | *Craugastor podiciferus* | UCR 22201 | Dota, San José, CR | 2395 | 9.7126 | -83.9488 | MK211634 | MK211590 | ––– |
| 23 | *Craugastor* sp. Chumacera | UCR 22120 | Buenos Aires, Puntarenas, CR | 1821 | 9.3218 | -83.4546 | MK211642 | ––– | SAMN43761716 |
| 24 | *Craugastor* sp. Chumacera | UCR 22690 | Pérez Zeledón, San José, CR | 1793 | 9.3267 | -83.4706 | MK211631 | MK211587 | ––– |
| 25 | *Craugastor* sp. Fila Costeña | EAP 0509 | Golfito, Puntarenas, CR | 1546 | 8.7878 | -83.0306 | ––– | MK211605 | SAMN43761717 |
| 26 | *Craugastor* sp. Fila Costeña | EAP 0519 | Pérez Zeledón, San José, CR | 1350 | 9.4415 | -83.6848 | ––– | ––– | SAMN43761718 |
| 27 | *Craugastor* sp. Fila Costeña | FMNH 257651 | Coto Brus, Puntarenas, CR | 1350 | 8.7833 | -82.9833 | EF562367 | ––– | ––– |
| 28 | *Craugastor* sp. Fila Costeña | UCR 16585 | Dota, San José, CR | 1400 | 9.5353 | -83.8580 | MK211647 | ––– | ––– |
| 29 | *Craugastor* sp. Fila Costeña | UCR 22091 | Pérez Zeledón, San José, CR | 1488 | 9.4410 | -83.6830 | MK211646 | MK211604 | ––– |
| 30 | *Craugastor* sp. Monte Verde | FMNH 257669 | Monte Verde, Puntarenas, CR | 1500 | 10.2773 | -84.5891 | EF562372 | MK211598 | ––– |
| 31 | *Craugastor* sp. Monte Verde | FMNH 257673 | Monte Verde, Puntarenas, CR | 1500 | 10.2773 | -84.5891 | EF562343 | MK211603 | ––– |
| 32 | *Craugastor* sp. Monte Verde | UCR 16361 | Alfaro Ruiz, Alajuela, CR | 1930 | 10.2176 | -84.3671 | EF562371 | ––– | ––– |
| 33 | *Craugastor* sp. Monte Verde | UCR 22675 | Puntarenas, Puntarenas, CR | 1726 | 10.3202 | -84.7987 | ––– | MK211606 | ––– |
| 34 | *Craugastor* sp. San Gerardo | CRARC 0247 | Tilarán, Guanacaste, CR | 1470 | 10.3600 | -84.8000 | MK211645 | ––– | ––– |
| 35 | *Craugastor* sp. San Gerardo | FMNH257671 | Monte Verde, Puntarenas, CR | 1500 | 10.2773 | -84.5891 | EF562374 | MK211599 | ––– |
| 36 | *Craugastor* sp. San Gerardo | UCR 16353 | Sarapiquí, Heredia, CR | 1500 | 10.2022 | -84.1625 | EF562349 | MK211602 | ––– |
| 37 | *Craugastor* sp. Pico Blanco | UCR 22226 | Escazú, San José, CR | 2242 | 9.8646 | -84.1429 | MK211644 | MK211601 | SAMN43761719 |
| 38 | *Craugastor* sp. Pico Blanco | UCR 22228 | Escazú, San José, CR | 2242 | 9.8646 | -84.1429 | MK211643 | MK211600 | ––– |
| 39 | *Craugastor* sp. Siola | EAP 0817 | Talamanca, Limón, CR | 1300 | 9.3987 | -83.0200 | MK211630 | MK211586 | ––– |
|  | *Craugastor stejnegerianus* clade | | | | | |  |  |  |
| 40 | *Craugastor gabbi* | UCR 22711 | Coto Brus, Puntarenas, CR | 1541 | 8.9515 | -82.8346 | OR419511 | OR420803 | SAMN43761720 |
| 41 | *Craugastor gabbi* | UCR 21863 | Coto Brus, Puntarenas, CR | 1200 | 8.7889 | -82.9583 | KT950271 | MK211567 | SAMN43761721 |
| 42 | *Craugastor gabbi* | UCR 21864 | Coto Brus, Puntarenas, CR | 1200 | 8.7889 | -82.9583 | KT950272 | MK211568 | SAMN43761722 |
| 43 | *Craugastor persimilis* | EAP 0586 | Talamanca. Limón, CR | 121 | 9.5773 | -82.9343 | MK211609 | MK211569 | SAMN43761723 |
| 44 | *Craugastor persimilis* | FMNH 257567 | Turrialba, Cartago, CR | 550 | 9.8917 | -83.6500 | ––– | ––– | SAMN43761724 |
| 45 | *Craugastor persimilis* | FMNH 257571 | Turrialba, Cartago, CR | 550 | 9.8917 | -83.6500 | OR419512 | OR420804 | ––– |
| 46 | *Craugastor persimilis* | UCR 22211 | Paraíso, Cartago, CR | 1050 | 9.7841 | -83.7517 | KT950293 | MK211570 | SAMN43761725 |
| 47 | *Craugastor persimilis* | UCR 22212 | Paraíso, Cartago, CR | 1050 | 9.7841 | -83.7517 | ––– | ––– | SAMN43761726 |
| 48 | *Craugastor rearki* | EAP 0554 | Upala, Guanacaste, CR | 764 | 10.7109 | -85.0406 | ––– | ––– | SAMN43761727 |
| 49 | *Craugastor rearki* | UCR 22640 | Upala, Guanacaste, CR | 764 | 10.7109 | -85.0406 | OR419513 | OR420805 | SAMN43761728 |
| 50 | *Craugastor rearki* | EAP 0572 | Siquírres, Limón, CR | 537 | 10.0595 | -83.5452 | ––– | ––– | SAMN43761729 |
| 51 | *Craugastor rearki* | MVZ 263735 | Altagracia, Altagracia, NI | 466 | 11.4687 | -85.5069 | OR419514 | OR420806 | ––– |
| 52 | *Craugastor rearki* | SMF 79759 | Matagalpa, Matagalpa, NI | 1300 | 12.9993 | -85.9092 | MK211608 | MK211565 | ––– |
| 53 | *Craugastor rearki* | UCR 20600 | Los Chiles, Alajuela, CR | 45 | 11.0513 | -84.7393 | OR419515 | OR420807 | SAMN43761730 |
| 54 | *Craugastor rearki* | UCR 16343 | Bagaces, Guanacaste, CR | 640 | 10.7072 | -85.0844 | OR419516 | OR420808 | ––– |
| 55 | *Craugastor rearki* | UCR 22240 | Pococí, Limón, CR | 228 | 10.2257 | -83.7712 | OR419517 | OR420809 | ––– |
| 56 | *Craugastor rearki* | UCR 21149 | Limón, Limón, CR | 400 | 9.9260 | -83.1880 | OR419518 | OR420810 | ––– |
| 57 | *Craugastor rearki* | UCR 21152 | Limón, Limón, CR | 400 | 9.9260 | -83.1880 | OR419519 | OR420811 | ––– |
| 58 | *Craugastor rearki* | USNM 559393 | Puerto Lempira, Gracias a Dios, HN | 190 | 14.9275 | -84.5339 | KU323364 | MK211566 | ––– |
| 59 | *Craugastor stejnegerianus* | EAP 0508 | Golfito, Puntarenas, CR | 28 | 8.6906 | -83.4815 | ––– | ––– | SAMN43761731 |
| 60 | *Craugastor stejnegerianus* | EAP 0512 | Buenos Aires, Puntarenas, CR | 812 | 9.0905 | -83.1247 | ––– | ––– | SAMN43761732 |
| 61 | *Craugastor stejnegerianus* | EAP 0514 | Osa, Puntarenas, CR | 45 | 8.9655 | -83.4411 | MK211607 | MK211563 | SAMN43761733 |
| 62 | *Craugastor stejnegerianus* | UCR 20346 | Buenos Aires, Puntarenas, CR | 900 | 9.0860 | -83.1110 | KT950283 | OR420812 | ––– |
| 63 | *Craugastor stejnegerianus* | UCR 20352 | Buenos Aires, Puntarenas, CR | 900 | 9.0863 | -83.1105 | KT950284 | MK211564 | ––– |
| 64 | *Craugastor stejnegerianus* | UCR 21494 | Golfito, Puntarenas, CR | 100 | 8.4046 | -83.1197 | KT950292 | OR420813 | ––– |
| 65 | *Craugastor stejnegerianus* | UCR 22070 | Buenos Aires, Puntarenas, CR | 416 | 9.1520 | -83.4256 | KT950281 | OR420814 | ––– |
| 66 | *Craugastor stejnegerianus* | UCR 22101 | Pérez Zeledón, San José, CR | 740 | 9.3009 | -83.7714 | ––– | OR420815 | ––– |
| 67 | *Craugastor stejnegerianus* | UCR 22280 | Osa, Puntarenas, CR | 30 | 9.1966 | -83.7870 | KT950280 | OR420816 | ––– |
| 68 | *Craugastor* sp. Neilly | UCR 22596 | Golfito, Puntarenas, CR | 182 | 8.6985 | -82.0489 | OR419520 | OR420817 | SAMN43761734 |
| 69 | *Craugastor* sp. Quepos | CRARC 0148 | San Ramón, Alajuela, CR | 1140 | 10.2021 | -84.4843 | OR419521 | OR420818 | SAMN43761735 |
| 70 | *Craugastor* sp. Quepos | CRARC 0266 | Montes de Oro, Puntarenas, CR | 1325 | 10.1800 | -84.6700 | OR419522 | OR420819 | ––– |
| 71 | *Craugastor* sp. Quepos | UCR 22609 | Aguirre, Puntarenas, CR | 12 | 9.3245 | -83.9498 | OR419523 | OR420820 | ––– |
| 72 | *Craugastor* sp. Quepos | EAP 0527 | Aguirre, Puntarenas, CR | 180 | 9.4776 | -84.0473 | ––– | ––– | SAMN43761736 |
| 73 | *Craugastor* sp. Quepos | UCR 20907 | Aguirre, Puntarenas, CR | 200 | 9.4619 | -84.0631 | OR419524 | OR420821 | ––– |
| 74 | *Craugastor* sp. Quepos | UCR 21007 | Aguirre, Puntarenas, CR | 200 | 9.4620 | -84.0630 | OR419525 | OR420822 | ––– |
| 75 | *Craugastor* sp. Quepos | UCR 22208 | Montes de Oca, San José, CR | 1210 | 9.9374 | -84.0495 | OR419526 | OR420823 | ––– |
|  | *Craugastor bransfordii* clade |  |  |  |  |  |  |  |  |
| 76 | *Craugastor bransfordii* | CRARC 0176 | Siquírres, Limón, CR | 755 | 10.0600 | -83.6300 | OR419527 | ––– | ––– |
| 77 | *Craugastor bransfordii* | EAP 0558 | Siquírres, Limón, CR | 537 | 10.0595 | -83.5452 | MK211610 | MK211572 | SAMN43761737 |
| 78 | *Craugastor bransfordii* | UCR 20559 | Los Chiles, Alajuela, CR | 45 | 11.0513 | -84.7393 | OR419528 | OR420824 | ––– |
| 79 | *Craugastor bransfordii* | UCR 20951 | San Ramón, Alajuela, CR | 1095 | 10.1862 | -84.5075 | OR419529 | OR420825 | ––– |
| 80 | *Craugastor bransfordii* | UCR 22269 | Alajuela, Alajuela, CR | 466 | 10.3121 | -84.1778 | KT950295 | MK211571 | SAMN43761738 |
| 81 | *Craugastor underwoodi* | EAP 0534 | Paraíso, Cartago, CR | 1412 | 9.7518 | -83.7792 | MK211611 | MK211573 | ––– |
| 82 | *Craugastor underwoodi* | EAP 0540 | Vázquez de Coronado, San José, CR | 1708 | 10.0254 | -83.9456 | MK211612 | MK211574 | SAMN43761739 |
| 83 | *Craugastor underwoodi* | UCR 22678 | Puntarenas, Puntarenas, CR | 1566 | 10.3160 | -84.8061 | OR419530 | OR420826 | SAMN43761740 |
| 84 | *Craugastor underwoodi* | CRARC 0245 | Tilarán, Guanacaste, CR | 1470 | 10.3600 | -84.8000 | OR419531 | ––– | ––– |
| 85 | *Craugastor* sp. Fila Carbon | EAP 0577 | Talamanca, Limón, CR | 94 | 9.7116 | -82.8344 | ––– | ––– | SAMN43761741 |
| 86 | *Craugastor* sp. Fila Carbon | EAP 0583 | Talamanca, Limón, CR | 198 | 9.6064 | -82.9115 | MK211613 | MK211575 | ––– |
| 87 | *Craugastor* sp. Fila Carbon | EAP 0585 | Talamanca, Limón, CR | 198 | 9.6064 | -82.9115 | ––– | ––– | SAMN43761742 |
| 88 | *Craugastor* sp. Fila Carbon | UCR 23149 | Talamanca, Limón, CR | 1500 | 9.3773 | -83.0371 | OR419532 | OR420827 | ––– |
| 89 | *Craugastor* sp. Fila Carbon | UCR 20050 | Talamanca, Limón, CR | 900 | 9.6178 | -83.2681 | MK211614 | MK211576 | ––– |
| 90 | *Craugastor* sp. Fila Carbon | UCR 20052 | Talamanca, Limón, CR | 900 | 9.6178 | -83.2681 | OR419533 | OR420828 | ––– |
| 91 | *Craugastor* sp. Fila Carbon | UCR 20150 | Limón, Limón, CR | 500 | 9.8550 | -83.1517 | OR419534 | OR420829 | ––– |
| 92 | *Craugastor* sp. Fila Carbon | UCR 21122 | Limón, Limón, CR | 400 | 9.9260 | -83.1880 | OR419535 | OR420830 | ––– |
| 93 | *Craugastor* sp. Fila Carbon | UCR 22533 | Limón, Limón, CR | 1123 | 9.8679 | -83.2406 | ––– | ––– | SAMN43761743 |
| 94 | *Craugastor* sp. Quebradas | UCR 16326 | Pérez Zeledón, San José, CR | 1313 | 9.4375 | -83.6869 | ––– | OR420831 | ––– |
| 95 | *Craugastor* sp. Panama | AJC 1921 | Panamá, Panamá, PA | 810 | 9.3198 | -79.2889 | KR863145 | KR862890. | ––– |
| 96 | *Craugastor* sp. Panama | AJC 1960 | Panamá, Panamá, PA | 624 | 9.2908 | -79.3027 | KR863147 | KR862892 | ––– |
| 97 | *Craugastor* sp. Panama | CH 6808 | Panamá, Panamá, PA | 871 | 9.2744 | -79.3178 | KR863146 | KR862891 | ––– |
| 98 | *Craugastor* sp. Panama | FMNH 257677 | Gualaca, Chiriquí, PA | 1000 | 8.7500 | -82.2167 | OR419536 | OR420832 | SAMN43761744 |
| 99 | *Craugastor* sp. Panama | FMNH 257698 | Kuna Yala, Kuna Yala, PA | 420 | 9.3167 | -78.9833 | ––– | ––– | SAMN43761745 |
| 100 | *Craugastor* sp. Panama | MVUP 1803 | La Pintada, Coclé, PA | 800 | 8.6670 | -80.5920 | FJ784358 | FJ766630 | ––– |
| 101 | *Craugastor* sp. Panama | MVUP 1841 | La Pintada, Coclé, PA | 800 | 8.6670 | -80.5920 | FJ784427 | FJ766628 | ––– |
| 102 | *Craugastor* sp. Panama | USNM 572220 | La Pintada, Coclé, PA | 800 | 8.6670 | -80.5920 | FJ784339 | FJ766631 | ––– |
| 103 | *Craugastor* sp. Panama | USNM 572221 | La Pintada, Coclé, PA | 800 | 8.6670 | -80.5920 | FJ784376 | FJ766629 | ––– |
| 104 | *Craugastor* sp. Panama | USNM 572222 | La Pintada, Coclé, PA | 800 | 8.6670 | -80.5920 | FJ784481 | FJ766627 | ––– |
| 105 | *Craugastor* sp. Panama | USNM 572223 | La Pintada, Coclé, PA | 800 | 8.6670 | -80.5920 | FJ784496 | FJ766626 | ––– |
| 106 | *Craugastor* sp. Panama | SMF 104010 | Changuinola, Bocas del Toro, PA | 1258 | 9.0090 | -82.6644 | OR419537 | OR420833 | SAMN43761746 |
| 107 | *Craugastor* sp. Vereh | CRARC 0047 | Turrialba, Cartago, CR | 1350 | 9.8681 | -83.4621 | OR419538 | OR420834 | SAMN43761747 |
| 108 | *Craugastor* sp. Vereh | CRARC 0048 | Turrialba, Cartago, CR | 1350 | 9.8681 | -83.4621 | OR419539 | OR420835 | ––– |
| 109 | *Craugastor* sp. Vereh | CRARC 0228 | Turrialba, Cartago, CR | 1650 | 9.7500 | -83.5500 | ––– | OR420836 | ––– |
| 110 | *Craugastor* sp. Vereh | UCR 23043 | Turrialba, Cartago, CR | 1650 | 9.7500 | -83.5500 | OR419540 | OR420837 | ––– |
