# Supplementary figures and images for: Deep cryptic diversity in the Craugastor podiciferus Species Group (Anura: Craugastoridae) of Isthmian Central America revealed by mitochondrial and nuclear data
Source: PeerJ. 2025 Jan 17;13:e18212. doi: 10.7717/peerj.18212 (PMC11745134; doi:10.7717/peerj.18212)

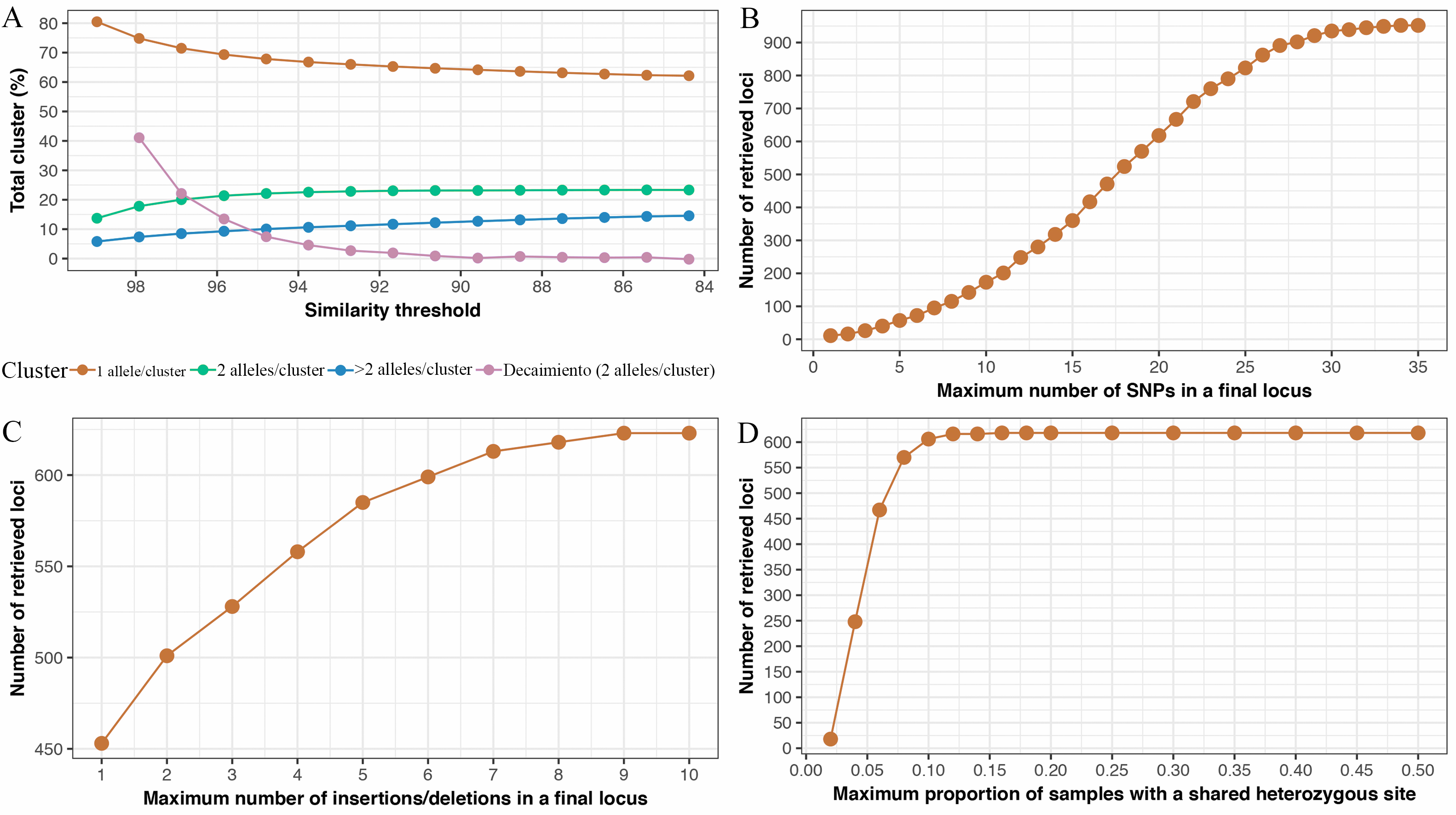

Supplement: Supplemental Information 2 — Results of multiples replicates of the seventh step of the software pipeline ipyrad v0.5.15 (Eaton, 2014), to determine the optimal value for the parameters: clustering parameter, maximum numbers of SNPs allowed in a locus, maximum number of insertions/deletions allowed in among-sample clusters, and the maximum proportion of samples allowed to share a heterozygous site. (A) Variation in the proportion of clusters with 1, 2, and ¿2 alleles retrieved with different similarity thresholds; (B) variation in the number of retrieved loci with different maximum numbers of SNPs in a final locus; (C) variation in the number of retrieved loci with different maximum numbers of insertions/deletions in across-sample clusters (c); (D) variation in the number of retrieved loci with different maximum proportions of samples with a shared heterozygous site. The different line colors in a represent different numbers of alleles/cluster, as indicated below the graph. [file peerj-13-18212-s002.png]
